# Telomere dysfunction implicates POT1 in patients with idiopathic pulmonary fibrosis

**DOI:** 10.1084/jem.20211681

**Published:** 2022-04-14

**Authors:** Joseph Kelich, Tomas Aramburu, Joanne J. van der Vis, Louise Showe, Andrew Kossenkov, Jasper van der Smagt, Maarten Massink, Angela Schoemaker, Eric Hennekam, Marcel Veltkamp, Coline H.M. van Moorsel, Emmanuel Skordalakes

**Affiliations:** 1 The Wistar Institute, Philadelphia, PA; 2 Department of Pulmonology, Interstitial Lung Disease Center of Excellence, St Antonius Hospital, Nieuwegein, Netherlands; 3 Department of Pharmacy and Biomedical Genetics, University Medical Center Utrecht, Utrecht, Netherlands

## Abstract

Exonic sequencing identified a family with idiopathic pulmonary fibrosis (IPF) containing a previously unreported heterozygous mutation in *POT1* p.(L259S). The family displays short telomeres and genetic anticipation. We found that POT1(L259S) is defective in binding the telomeric overhang, nuclear accumulation, negative regulation of telomerase, and lagging strand maintenance. Patient cells containing the mutation display telomere loss, lagging strand defects, telomere-induced DNA damage, and premature senescence with G1 arrest. Our data suggest POT1(L259S) is a pathogenic driver of IPF and provide insights into gene therapy options.

## Teaser

This study reports the first known family with pulmonary fibrosis carrying a heterozygous *POT1* mutation. The family displays short telomeres, short telomere phenotypes, and genetic anticipation. The data suggest POT1(L259S) is a pathogenic driver of IPF and provide insights into gene therapy options.

## Introduction

Telomeres protect chromosome ends from erroneous DNA-damage response (DDR) and solve the end replication problem (O’Sullivan and Karlseder, 2010). With each round of cell division, telomeres become progressively shorter until reaching a critical length whereupon senescence or apoptosis is initiated (Blasco, 2005). Telomeres are subject to tight regulation within the cell that is essential to organismal health. Aberrant shortening or elongation of telomeres poses risks for genomic instability, stem cell failure, and cancer (Blasco, 2005). Various protein complexes are responsible for maintaining proper telomere homeostasis, including the telomerase holoenzyme, shelterin, and the CTC1, Stn1, Ten1 protein (CST) complex (Lim and Cech, 2021).

Shelterin is a six-protein complex (consisting of TRF1, TRF2, RAP1, TIN2, POT1, and TPP1) that recruits telomerase to telomeres for telomere elongation and caps the ends of chromosomes (Palm and de Lange, 2008). Chromosome end-capping suppresses ataxia telangiectasia mutated (ATM)– and ataxia telangiectasia and Rad3-related (ATR)–dependent DDR pathways, preventing illicit activation of DNA repair mechanisms at telomeres, such as nonhomologous end joining and homology-directed repair (Palm and de Lange, 2008). In the absence of shelterin, these repair mechanisms result in chromosomal end-to-end fusions, telomere sister chromatid exchanges, and the formation of unstable telomere-free chromosome ends (Rai et al., 2016; van Steensel et al., 1998).

POT1-TPP1, a subunit of shelterin, is critical to both telomere capping and length regulation. The N-terminus of POT1 (POT1N) consists of two OB folds and binds telomeric single-stranded DNA with high affinity and specificity (Lei et al., 2003; Lei et al., 2004). The POT1 C-terminus (POT1C) comprises an oligonucleotide/oligosaccharide-binding fold (OB3) and a Holliday junction resolvase domain, both of which are involved in TPP1 binding, the shelterin component that recruits telomerase to telomeres (Rice et al., 2017; Chen et al., 2017). POT1 has additionally been shown to resolve G-quadruplexes that are formed in telomeric DNA (Zaug et al., 2005; Chaires et al., 2020). G-quadruplexes are secondary structures that can prevent proper telomere maintenance. POT1 is thought to have functions in both leading and lagging strand synthesis (Arnoult et al., 2009; Wang et al., 2012; Chen et al., 2012). The POT1–TPP1 complex recruits telomerase to the telomeric overhang for telomere elongation (Wang et al., 2007). POT1’s role in lagging strand replication is less understood but may involve recruitment of the CST complex (which facilitates lagging strand fill-in via DNA polymerase α primase; Giraud-Panis et al., 2010; Gu et al., 2021; Takai et al., 2016). Loss of POT1 is embryonically lethal and is associated with rapid ATR-dependent DDR, elongated telomeres, and cell cycle arrest (Wu et al., 2006).

Mutations in POT1–TPP1 have been implicated in certain telomere syndromes, such as Coats plus (CP), dyskeratosis congenita, bone marrow failure, liver disease, and idiopathic pulmonary fibrosis (IPF). Heterozygous carriage of *TPP1* variants are associated with dyskeratosis congenita, bone marrow failure, and IPF (Guo et al., 2014; Hoffman et al., 2019), while homozygous carriage of a *POT1* variant has been implicated in CP (Takai et al., 2016). To our knowledge, *POT1* mutations have not been previously reported in IPF pathology.

Mutations within key telomeric genes, including *TERT*, *TERC*, *DKC1* (dyskerin), *RTEL1*, *PARN*, and *TINF2* (TIN2) have been implicated in IPF, a chronic disease affecting the lungs (Stuart et al., 2015; Hoffman et al., 2016). IPF occurs by progressive scarring (fibrosis) within the lungs with accumulation of scar tissue leading to decreased oxygen uptake, respiratory failure, and mortality (Martinez et al., 2017). Currently, IPF confers poor prognosis, with mean survival placed 3–5 yr following diagnosis (Gulati and Luckhardt, 2020). While the exact cause of IPF is relatively unknown, there are a range of known contributing factors including environmental exposures, aging, and telomere dysfunction (Barratt et al., 2018). Strikingly, ≤25% of familial IPF and ≤3% of sporadic IPF patients have mutations in telomeric complexes (Armanios and Blackburn, 2012). It is current opinion that exogenous damage to lung tissues combined with critically short telomeres catalyzes the onset of IPF (Armanios and Blackburn, 2012).

Here we report the first known family with pulmonary fibrosis carrying a heterozygous *POT1* mutation. Four patients, treated for IPF, are carriers of the *POT1* p.(L259S) mutation. Patients were found to have short telomeres in peripheral blood. Interestingly, the POT1(L259S) mutant protein is defective in DNA binding. Correspondingly, we found that fibroblasts obtained from patient V-1 carrying this mutation have decreased nuclear POT1, significant telomere loss, and increased telomere dysfunction–induced foci. These defects arising from POT1(L259S) lead to growth defects and increased senescence. Taken together, these data demonstrate a possible mechanism responsible for causing IPF in patients carrying a heterozygous mutation in *POT1*.

## Results

### Identification of a novel POT1 mutation in familial IPF

**Figure 1.**
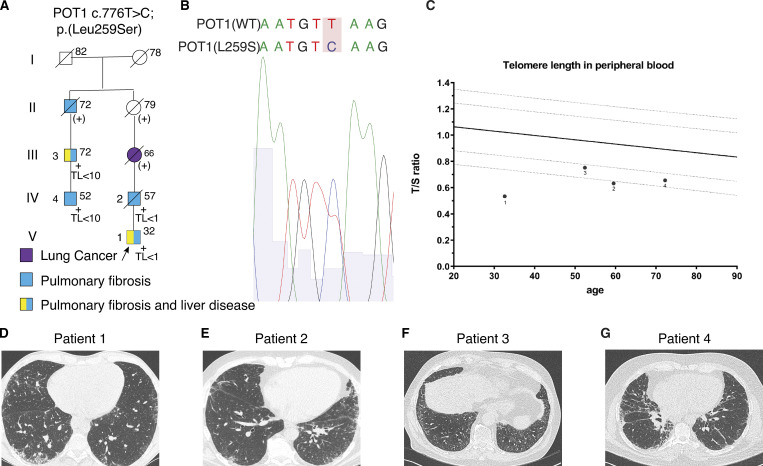
**POT1(L259S) variant patient data. (A)** Abridged pedigree of family with *POT1* c. 776T>C mutation. Presence of the *POT1* c.776T>C; p.(Leu259Ser) gene mutation is indicated by +. (+), presumed carriage of the familial mutation with no DNA available. Circles represent females; squares represent males; symbols with a slash represent deceased individuals. Arrow refers to the proband, roman numerals indicate the generation, and numbers at the left side of the symbols represent the patient numbering in the article. Age at time of diagnosis or age of death is indicated at the upper right of each symbol; TL, telomere length in peripheral blood leukocytes measured by quantitative PCR; TL <1, lower than first percentile of control subjects; TL <10, lower than 10th percentile of control subjects. **(B)** Patient fibroblast genomic DNA Sanger sequencing shows the heterozygous mutation 776T>C. **(C)** Telomere length of patients 1–4 measured by a quantitative PCR assay plotted against age. The solid line indicates the 50th percentile of control subjects; the lower dotted lines indicate the first and 10th percentiles of control subjects. **(D)** HRCT scans of the chest for patient 1 at first visit, classified as probable UIP (pUIP). **(E)** Patient 2 HRCT at first visit, classified as pUIP pattern. **(F)** Patient 3 HRCT at first screening visit, classified as pUIP pattern. **(G)** Patient 4 HRCT at first visit, showing a pUIP pattern.

The first patient (patient 1, proband pedigree V-1; Fig. 1 A) was a 32-yr-old male referred to our center because of features of interstitial lung disease on high-resolution computed tomography (HRCT). He had been diagnosed with nodular regenerative hyperplasia of the liver at age 23. Furthermore, he had portal hypertension with esophagus and fundus varices and splenomegaly with splenic vein thrombosis and thrombocytopenia. His family history was notable for IPF in his father. He had a smoking history of 7 packs/yr. On physical examination, digital clubbing was noted. Pulmonary fibrosis, with a probable usual interstitial pneumonia (UIP) pattern, was seen on HRCT, and the patient was given a diagnosis of IPF (Fig. 1 D). He was treated with pirfenidone for 17 mo and switched to nintedanib because of progression of disease and side effects. Treatment with nintedanib was discontinued after 8 mo due to side effects. At age 35, he underwent liver transplantation due to hepatopulmonary syndrome. 6 mo after liver transplantation, treatment with danazol was started. Pulmonary fibrosis was progressive, and 2 yr after liver transplantation, he underwent lung transplantation. Next-generation sequencing on a 36-gene IPF gene panel revealed a heterozygous variant in *POT1*: c.776T>C; p.(Leu259Ser) (NM015450.2_) and no abnormalities in the other 35 genes on the panel. The L259S variant was inherited from the deceased father with IPF and confirmed to be present in extracted patient fibroblasts with Sanger sequencing (Fig. 1 B). Telomere length in blood showed short telomeres, below the first percentile of controls (Fig. 1 C).

The second patient was a 57-yr-old male (pedigree IV-2), the father of patient 1, who presented with dyspnea on exertion. His past medical record revealed peritoneal fibrosis. He had a smoking history of 15 packs/yr. On physical examination, bilateral respiratory crackles were noted. Pulmonary fibrosis with a probable UIP pattern was seen on HRCT (Fig. 1 E). He was diagnosed with IPF and treated with pirfenidone for 1 yr. Treatment was stopped because of persistent progression of disease, and 1 mo later the patient died at age 60. Targeted genetic analysis revealed a heterozygous variant in *POT1*: c.776T>C; p.(Leu259Ser).

Patient 3 (pedigree III-3) was a 72-yr-old male referred for a second opinion. He had dyspnea on exertion for several years and reported graying of his hair at age 27. He presented with a probable UIP pattern on chest HRCT, thrombocytopenia, and RBC macrocytosis (Fig. 1 F). His medical history revealed a partial cholecystectomy due to fibrosing cholecystitis and liver abnormalities. He had a history of smoking shag tobacco for 27 yr. His family history was notable for IPF in his son and his father, who had a UIP pattern on biopsy. He was treated with nintedanib for 6 mo, and treatment was discontinued due to side effects. Genetic analysis revealed a heterozygous variant in *POT1*: c.776T>C; p.(Leu259Ser) and no abnormalities in any of the other 35 genes on the IPF gene panel.

Patient 4 (pedigree IV-4) was a 52-yr-old-male, son of patient 3, who presented with dyspnea and progressive dry cough for 1 yr. He had no history of smoking. On physical examination, bibasal crepitations and digital clubbing were noted. He presented with a probable UIP pattern on chest HRCT (Fig. 1 G). He was diagnosed with IPF and was treated with nintedanib. After 1 yr of treatment, additional danazol was prescribed. Genetic analysis on our 36-gene pulmonary fibrosis gene panel revealed a heterozygous variant in *POT1*: c.776T>C; p.(Leu259Ser) and no abnormalities in the other 35 genes tested.

Genealogical research revealed that patient 3 is a maternal cousin of patient 2 (Fig. 1 A). Demographics and clinical characteristics at first visit of patients 1–4 are presented in Table 1. The POT1(L259S) missense substitution was classified as a variant of uncertain clinical significance. The variant is not present in the gnomAD database or other population frequency databases. The leucine residue represents a moderately conserved amino acid, and the serine residue is found in platypus and chicken. The leucine-to-serine substitution does represent a large physicochemical difference. Interestingly, missense prediction algorithms (Align GVGD, SIFT, MutationTaster, and Polyphen-2) mostly predict the variant as a benign variant. Because the mutation is newly discovered and has unknown significance, we sought to experimentally demonstrate the pathogenic effect of the mutation.

**Table 1.** Demographics and clinical characteristics of patients 1–4 with IPF

Characteristic	Patient 1	Patient 2	Patient 3	Patient 4
Sex	M	M	M	M
Age at diagnosis (yr)	32	57	72	52
FVC (% predicted)	89	78	102	62
DLCOc (% predicted)	40	65	75	47
Smoking behavior	Former	Former	Former	Never
Pack years	7	27	30	NA
HRCT pattern	Probable UIP	Probable UIP	Probable UIP	Probable UIP
Inspiratory crackles	Yes	Yes	No	Yes
Digital clubbing	No	Yes	No	No
Additional STS	Liver disease	Peritoneal fibrosis	Liver disease; thrombocytopenia, RBC macrocytosis	No
T/S_obs-exp_	−0.488	−0.301	−0.236	−0.204
T/S <10th percentile	Yes	Yes	Yes	Yes

DLCOc, diffusing capacity of the lungs for carbon monoxide corrected for hemoglobin concentration; FVC, forced vital capacity; STS, short telomere syndrome; T/S_obs-exp_, telomere length adjusted for age calculated by the difference between observed T/S ratio and the age-adjusted normal value.

### The POT1(L259S) mutant protein is defective of DNA binding

**Figure 2.**
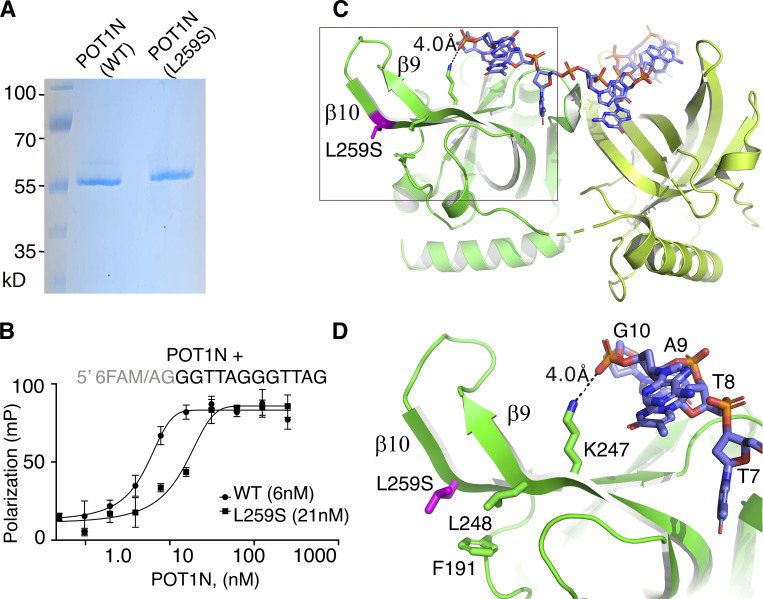
**POT1(L259S) binds DNA with reduced affinity. (A)** SDS-PAGE gel of WT and mutant POT1N. **(B)** FP assays of WT and mutant POT1N. The data show that POT1(L259S) binds telomeric DNA with ∼3.5-fold reduced affinity compared with the WT protein. mP, milipolarization. **(C)** Structure of POT1N (green), DNA (blue/orange; PDB ID: 1XJV). **(D)** Zoom in at the second OB fold of POT1N and where the mutation L259S is located; same residue and nucleotide numbering as in Lei et al. (2004). Residues (L248, F191) involved in contacts with L259S are shown. Also, K247, which makes a weak hydrogen bond with the backbone of the telomeric DNA, is shown. Source data are available for this figure: SourceData F2.

The L259S mutation localizes to the N-terminal portion of POT1 involved in telomeric DNA binding (Lei et al., 2003; Lei et al., 2004; Nandakumar and Cech, 2012). Therefore, we asked whether the L259S mutation could affect POT1-DNA association. To address this question, we expressed and purified to homogeneity the WT and mutant (L259S) POT1 N-terminal OB folds (residues 6–299; Fig. 2 A). The proteins were overexpressed with a hexahistidine and a fusion tag (Ocr protein from bacteriophage T7) N-terminally to the POT1N proteins for purification and solubility purposes.

To determine the DNA binding affinity of the two POT1N (WT and L259S) proteins, we performed fluorescence polarization (FP) assays. FP assays were carried out using a previously identified POT1 DNA binding probe containing a fluorescein tag at the 5′ end (6-FAM/5′-AGGGTTAGGGTTAG-3′). Two extra nucleotides were added to the 5′ end of the DNA probe because the fluorescence of 6-FAM is reduced when next to a guanine. The FP data indicate that WT POT1N binds to the telomeric overhang with a *K*_*d*_ of 6 ± 2 nM (Fig. 2 B), consistent with what has been previously reported (Lei et al., 2004). Interestingly, we found that the POT1N(L259S) mutant protein exhibits a significant decrease in DNA binding affinity, approximately 3.5× that of the WT POT1 (*K*_*d*_ = 21 ± 6 nM). The P value <0.0001 was determined from six independent experiments, each run in triplicate.

To further understand how the POT1(L259S) mutant affects DNA binding, we reverted to the published POT1N-DNA structure (Lei et al., 2004). L259S is part of a cluster of hydrophobic residues consisting of L248 and F191. This hydrophobic cluster is critical to the formation and organization of the POT1N hairpin formed by the β strands β9 and β10 (Fig. 2, C and D). The hairpin in turn forms part of the canonical OB binding pocket of OB2 of POT1 and is involved in telomeric DNA binding. Specifically, residue K247 is within coordinating distance (4.0 Å) of the telomeric DNA. It forms a weak hydrogen bond with the hydroxyl of the phosphate group of the guanine located at the 3′ end of the DNA probe. Introduction of a polar amino acid in the location of a nonpolar, aliphatic residue most likely disrupts the organization of the hairpin and the interaction of K247 with the DNA, leading to loss of DNA binding affinity observed in the FP assays.

### POT1(L259S) localizes to telomeres

**Figure 3.**
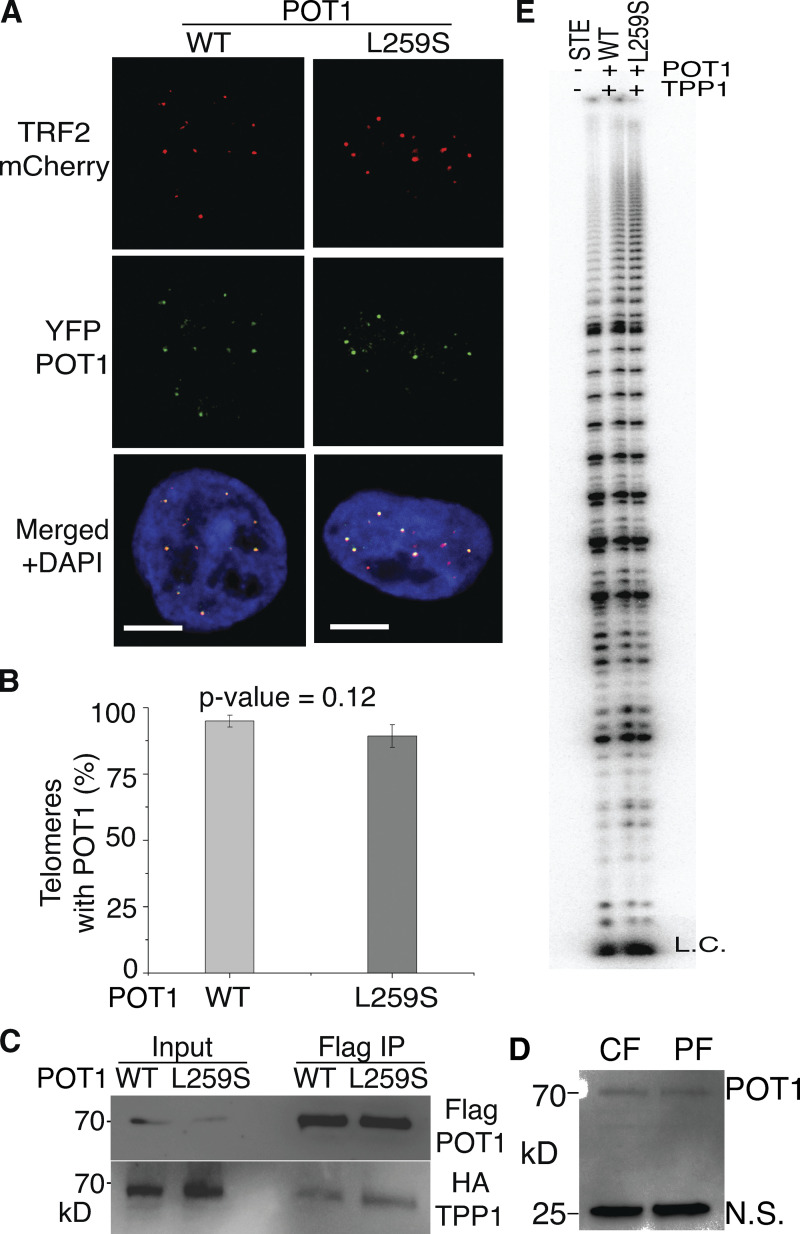
**POT1(l259S) localizes to telomeres, binds TPP1, and does not affect telomerase activity/processivity. (A)** Confocal microscopy images are shown of HEK293T cells expressing YFP-POT1 as WT or L259S. mCherry-TRF2 was coexpressed to label telomeres (scale bar, 5 μm). **(B)** Quantification of the percentage of telomeric spots colocalized with POT1. **(C)** Coimmunoprecipitations were performed using Flag beads to pull out Flag-POT1. Ability of mutant POT1 and WT POT1 to bind HA-TPP1 was addressed with anti-HA. **(D)** Western blot using anti-POT1 antibodies to probe lysates from control fibroblasts (CF) and patient fibroblasts (PF). N.S., nonspecific band used as a loading control. Lysates show similar amounts of POT1 between POT1(WT)- and POT1(L259S)-containing samples of control and patient fibroblasts, respectively. **(E)** Direct telomerase primer extension assays were conducted with super telomerase extract expressing hTERT and hTR alone (STE) and with POT1(WT)/TPP1 and POT1(L259S)/TPP1. The L259S mutant has no obvious effect on telomerase activity or processivity compared with WT. Source data are available for this figure: SourceData F3.

We then asked whether POT1(L259S) localizes to telomeres in cells. Because TPP1 is known to increase the binding affinity of POT1 to telomeres (Rice et al., 2017; Wang et al., 2007; Xin et al., 2007), we tested whether POT1(L259S) still interacts with TPP1 in cells. Using Flag-POT1 coimmunoprecipitations for WT and L259S POT1, we found that POT1(L259S) can still interact with TPP1 with similar efficiency (Fig. 3 C). To further address whether POT1(L259S) is defective in telomeric localization, we introduced YFP-POT1 WT and L259S into HEK293T cells stably knocked down for endogenous POT1 as in our previous publication (Rice et al., 2017). mCherry-TRF2 was also introduced as a telomeric marker. Transfected cells expressing YFP-POT1 (WT or L259S) and mCherry-TRF2 were then grown on microscope slides and imaged with confocal microscopy. YFP-POT1 (WT) or (L259S), mCherry-TRF2 colocalization measurements from >120 telomeres show a slight decrease (∼5%) in POT1(L259S) localization at telomeres compared with WT (Fig. 3 A), results that are not statistically significant. This indicates that decrease in telomeric binding affinity of POT1(L259S) does not drastically change its ability to localize to telomeres within cells. This is not surprising, since POT1 recruitment to telomeres depends on TPP1, an interaction not affected by the L259S mutant (Ye et al., 2004). Even through POT1(L259S) localizes to telomeres in a manner similar to WT, the amount of POT1 molecules loaded on the actual single-stranded overhang may be decreased.

### POT1(L259S) does not significantly affect telomerase activity or processivity

We next asked whether POT1(L259S) negatively affects telomerase processivity. Because patients containing the L259S variant had short telomeres, we questioned whether this was a consequence of reduced telomerase processivity. To test this hypothesis, we generated super telomerase extracts as described in previous publications (Rice et al., 2017; Cristofari and Lingner, 2006), supplemented with either POT1(L259S) or WT as well as TPP1. HEK293T cells were cotransfected with plasmids containing hTERT, hTR, POT1 (L259S or WT), and TPP1. Direct telomerase human telomerase activity assays do not show any significant differences in telomerase processivity between POT1(WT) and L259S (Fig. 3 D). These data show that the L259S variant does not negatively affect telomerase extension activity in vitro.

### The POT1(L259S) mutant protein has reduced nuclear localization

**Figure 4.**
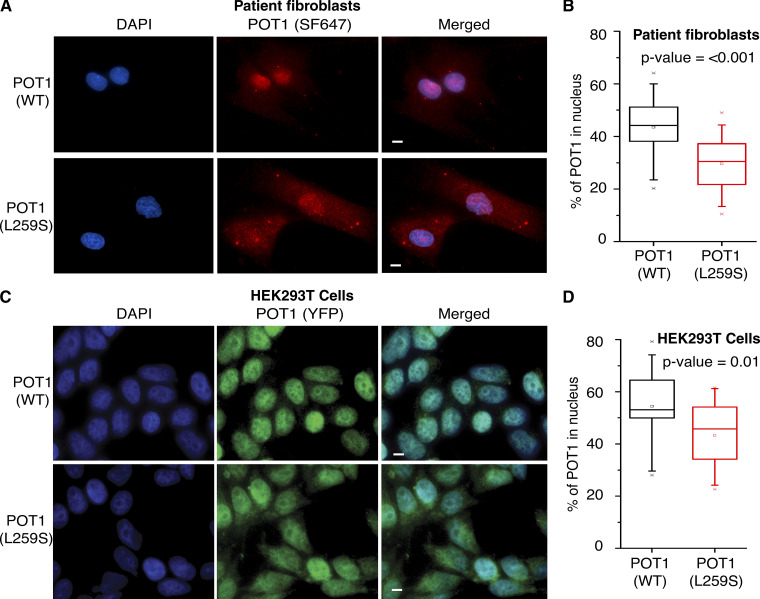
**POT1(L259S) is defective in nuclear localization. (A)** Representative images are shown for control and patient-obtained fibroblasts. DAPI is used for a nuclear stain, and SF647 was used in combination with anti-POT1 antibodies (scale bar, 5 μm). **(C)** Representative images are shown for YFP-POT1 WT– and L259S-expressing HEK293T cells (scale bar, 5 μm). **(B and D)** Nuclear/cytoplasmic ratio was quantified for data in A and C. Data were obtained from two independent experiments, each performed in duplicate.

Because we have determined that POT1(L259S) is defective of DNA binding, we asked whether this mutation leads to defective intracellular localization. To assess this question, we obtained normal diploid fibroblasts (CCD-1058Sk, American Type Culture Collection CRL-2071; control) and the POT1(L259S)-bearing IPF patient fibroblasts. After growing these cells in parallel, we conducted immunofluorescence imaging staining for POT1 to observe the percentage of POT1 within the nucleus and cytoplasm using DAPI nuclear stain and secondary antibody Santa Cruz Fluorophore 647 probed POT1. Nuclear/cytoplasmic percentages of POT1 (WT and L259S) were then calculated using Intensity_Ratio_Nuclei_Cytoplasm.ijm software (Kelich et al., 2021). Remarkably, we found that POT1(L259S) containing fibroblasts exhibit a marked reduction in nuclear POT1 compared with control cells (Fig. 4, A and B; P < 0.001). We analyzed 120 and 80 cells for healthy and IPF samples, respectively. We found that 43.45% of intracellular POT1(WT) localized to the nucleus in healthy cells and 29.68% of POT1(L259S) in IPF patient-derived cells. This finding indicates a reduction in nuclear POT1(L259S) and a corresponding increase in cytoplasmic POT1. To confirm these results in isogenic controls, we introduced YFP-POT1(L259S) and YFP-POT1(WT) in HEK293T cells knocked down for endogenous POT1 and completed the same analysis as was conducted for the patient-obtained and healthy fibroblasts. After analyzing 279 and 341 cells for WT and L259S-expressing cells, respectively, we report an 11% decrease in nuclear POT1 for L259S relative to WT (Fig. 4, C and D). These data confirm that POT1(L259S) results in a decrease in nuclear localization.

### Patient fibroblasts harboring the POT1(L259S) variant have shortened lagging strands

We then set out to examine the effects of the POT1(L259S) mutation on telomere homeostasis. To address whether the POT1(L259S) mutation affects leading and lagging strand synthesis, we used interphase telomere-FISH. This technique allows for fluorescence quantification of telomeric signal. We used two separate peptide nucleic acid (PNA) probes, targeting the leading and lagging telomeric strands (PNA-Bio). We used the signal obtained from these studies to calculate the signal ratio of leading versus lagging strands and to see if either strand is disproportionally shortened or lost.

**Figure 5.**
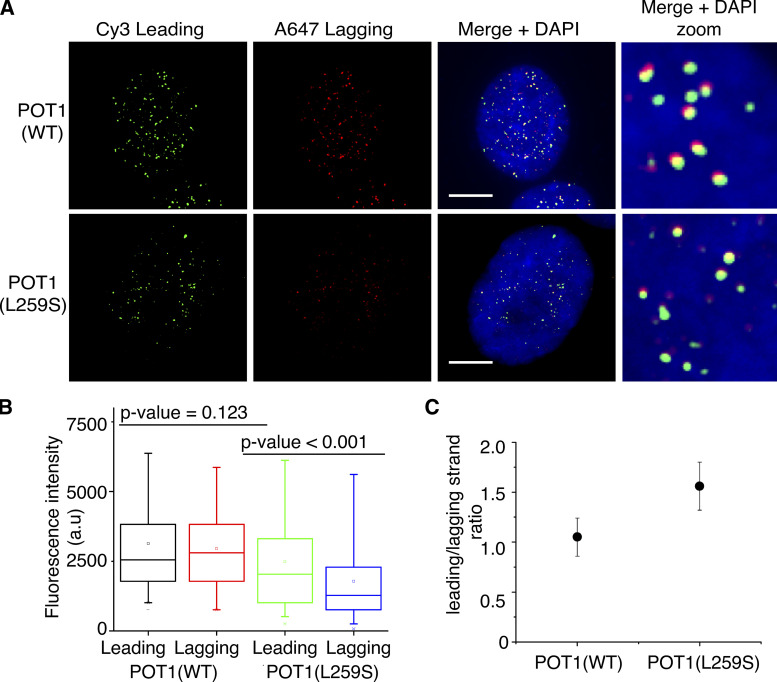
**Fibroblasts containing L259S have shorter telomeres and greater leading/lagging strand ratio. (A)** Representative images are shown displaying chromosome orientation (CO)–FISH probing of leading and lagging strand. Cy3 was used for leading strand, and Alexa Fluor 647 was used for lagging strand. DAPI was used for nuclear staining (scale bar, 5 μm). **(B)** Quantification of fluorescence intensity for both leading and lagging strands. Data were obtained from two independent experiments, each performed in duplicate. **(C)** Leading/lagging strand fluorescence intensity ratio was quantified for control fibroblasts and patient-obtained samples.

Our telomere-FISH experiments measuring 374 and 186 telomeric spots for IPF and control cells, respectively, revealed that fibroblasts containing POT1(L259S) have an increased leading/lagging strand ratio compared with healthy fibroblasts (Fig. 5). Through this technique, we saw a modest decrease in intensity for the leading strands of POT1(L259S) cells compared with healthy ones. However, we witnessed a near-twofold decrease in lagging strand fluorescence intensity (P < 0.001) between the cells carrying the WT and those carrying the POT1(L259S) variant. We then analyzed the ratios between leading and lagging strands for each condition. The ratio obtained for POT1(L259S) was 1.56, and the ratio for the control fibroblasts was only 1.06, indicating a disproportionally shortened lagging strand (P < 0.001) in IPF patient cells.

### POT1(L259S) causes an increase in telomeric overhang signal and is defective in regulating telomere length

**Figure 6.**
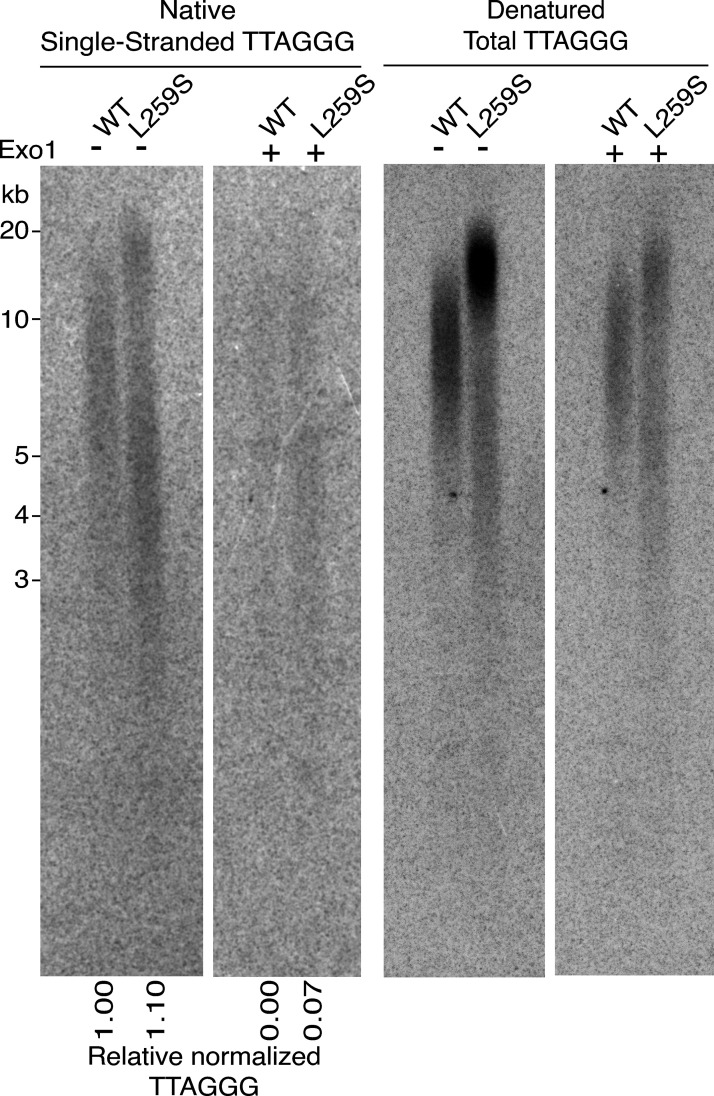
**3′ overhang and total telomere signal analysis of HEK293T cells expressing POT1 (L259S) and POT1(WT).** Southern blots conducted on genomic DNA extracted from HEK293T cells expressing POT1(WT) or POT1(L259S) for 60 d are shown. The gel was probed for TTAGGG signal in both native conditions (to detect single-stranded DNA) and denaturing conditions (to detect total telomeric signal). DNA was also treated with Exo1 to digest 3′ ends and detect internal single-stranded DNA (Exo1 resistant). The relative normalized telomeric signal intensity for single-stranded TTAGGG signal is shown below. Source data are available for this figure: SourceData F6.

To further understand how POT1(L259S) is defective in telomere maintenance, we performed analysis of the single-stranded 3′ telomeric overhang on HEK293T cells expressing POT1(L259S) or POT1(WT) for 60 d. Use of nondenaturing in-gel Southern blotting to hybridize CCCTAA probes with genomic DNA can reveal the abundance of single-stranded telomeric signal. By normalizing the single-stranded signal to the total telomeric signal obtained from subsequent denaturing gel conditions, we quantified the abundance of single-stranded telomeres. Also, by treating the DNA with Exo1, we confirmed the 3′ end nature of the probed telomeres and detected the portion of Exo1 DNA that is indicative of internal stretches of DNA. We found that HEK239T cells expressing POT1(L259S) had 10% more relative single-stranded overhang signal than cells expressing POT1(WT) (Fig. 6). We also detected an ∼6% increase in Exo1-resistant telomeric DNA for POT1(L259S) compared with POT1(WT). Moreover, HEK293T cells expressing POT1(L259S) developed longer telomeres compared with POT1(WT)-expressing cells, as shown from the denatured Southern blot (Fig. 6, right panel). This suggests that POT1(L259S) is defective in limiting telomere elongation within cells.

### POT1(L259S) causes loss of telomeric signal in metaphase chromosomes

**Figure 7.**
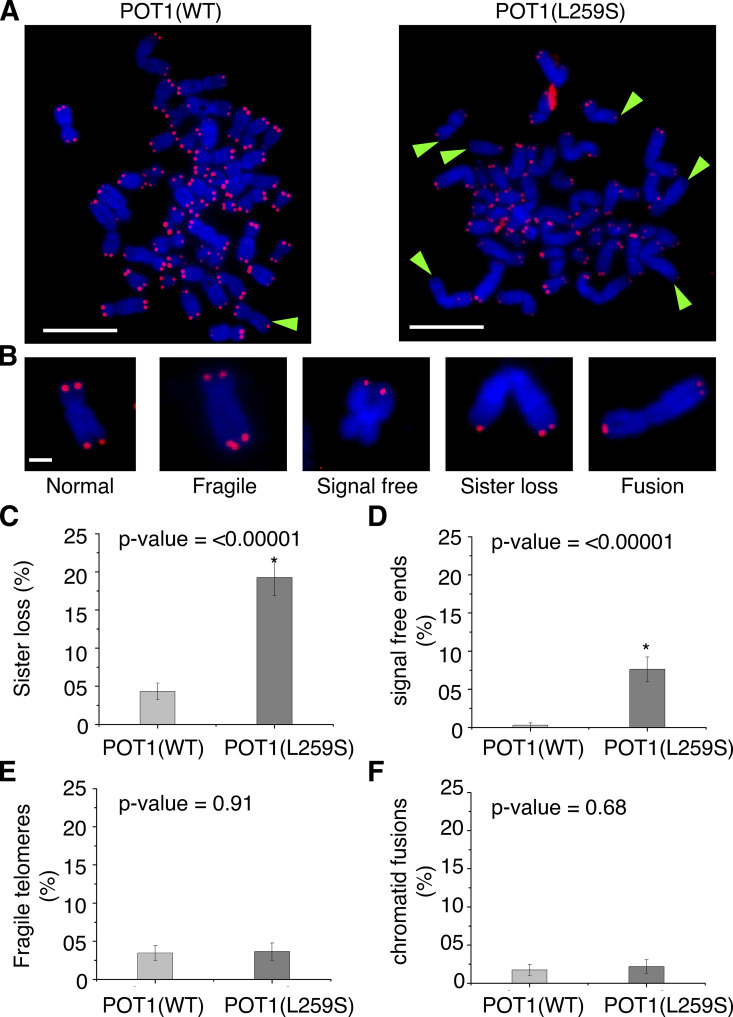
**Metaphase spreads reveal missing telomeres in POT1(L259S) fibroblasts. (A)** Representative metaphase spreads for control and patient-obtained fibroblasts (scale bar, 5 μm). Both populations were split onto slides at six population doublings from the initial stock. Green arrowheads show missing telomeres. Blue channel is DAPI, while red channels are PNA-TELO probes labeled with Alexa Fluor 647. Data for each panel were obtained from two independent experiments, each performed in duplicate. **(B)** Representations of various telomeric defects found in samples (scale bar, 1 μm). **(C)** Quantification of the percentage of chromosomes with missing sister telomeres. **(D)** Quantification of chromosomes with signal-free ends. **(E)** Percentage of chromosomes with fragile telomeres. **(F)** Percentage of chromosomes with chromatid fusions.

We then examined the POT1(L259S) cells for telomeric abnormalities such as sister telomere loss, complete loss of telomeric signal (signal-free ends), fragile telomeres, and telomeric fusions, frequently associated with defective telomeric complexes, using PNA-FISH. To screen for these defects, we generated metaphase spreads of chromosomes from control- and POT1(L259S) variant–containing fibroblasts. Remarkably, we found a significant (P < 0.0001) increase in sister telomere loss for POT1(L259S)-containing cells compared with control cells (19 and 4%, respectively; Fig. 7). Additionally, no signal-free telomeric losses were witnessed for control cells, whereas 8% of the chromosomes from POT1(L259S)-containing cells had signal-free ends. No significant differences were found for control and IPF patient fibroblasts for fragile telomeres and telomeric fusions.

### POT1(L259S) leads to increase in telomere dysfunction–induced foci (TIFs)

**Figure 8.**
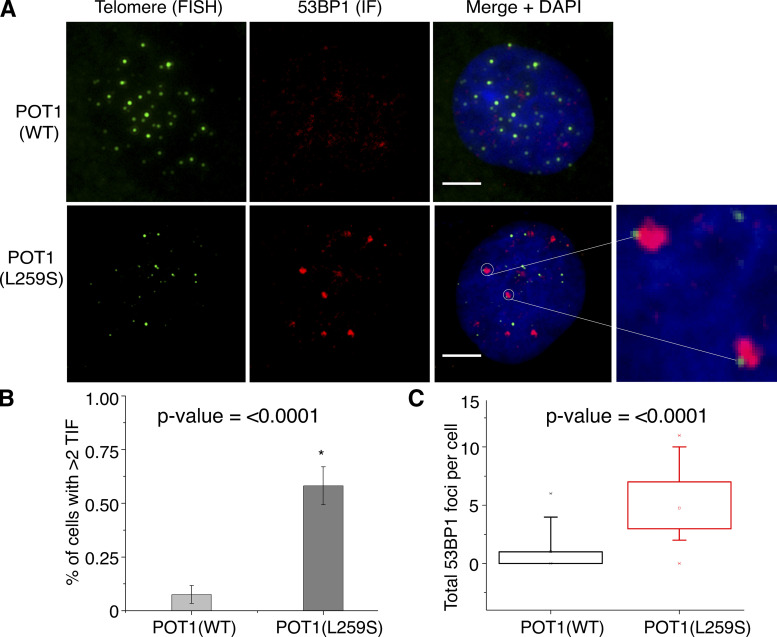
**53BP1 immunofluorescence reveals increased TIF for POT1(L259S) fibroblasts. (A)** Representative images are shown for both control and L259S-containing fibroblasts (scale bar, 5 μm). The green channel represents PNA-FISH probing for telomeric repeats. The red channel shows the immunofluorescence data for 53BP1 antibody staining. The blue color represents DAPI nuclear staining. Colocalizations between telomeres and 53BP1 identified TIFs as shown highlighted by white circles and the adjacent zoomed images. **(B)** Quantifications are shown for the percentage of cells with at least two TIF signals. Data were obtained from two independent experiments, each performed in duplicate. **(C)** Total 53BP1 foci were quantified for patient fibroblasts containing POT1(L259S) and control fibroblasts.

Our findings led us to speculate that telomeres of patients harboring the POT1(L259S) mutation may not be as efficiently capped as control cells with POT1(WT). This would lead to an inability to repress DDR at telomeres, which can induce TIFs. To screen for TIFs, we used a combination of telomeric FISH and immunofluorescence staining for the DDR factor 53BP1. From analysis of 40 and 30 cells for control and IPF-derived fibroblasts, respectively, we found that although only 7.5% of control POT1(WT) fibroblasts had at least two TIFs, this percentage rose to 58% for POT1(L259S)-containing patient fibroblast cells (P < 0.0001; Fig. 8). The total 53BP1 foci was also drastically higher for patient fibroblasts. This is indicative that IPF patients harboring POT1(L259S) are defective in telomere capping, resulting in an increase of TIFs. It is worth noting that TIFs are primarily associated with weaker telomeric signals, potentially suggesting that TIFs are associated with shorter telomeres of IPF patients.

### POT1(L259S) induces premature senescence

**Figure 9.**
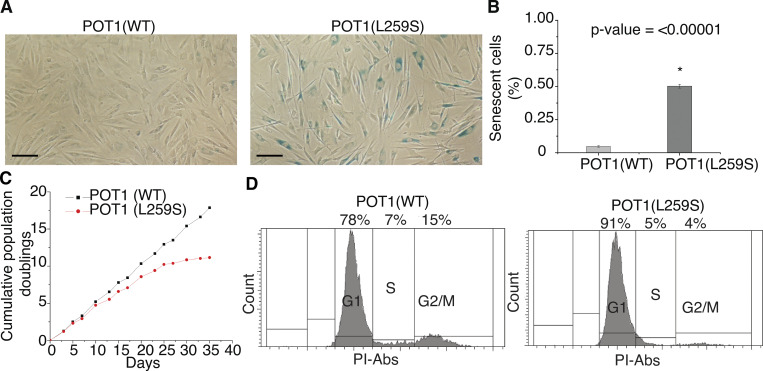
**Fibroblasts carrying the POT1(L259S) display a high proportion of senescence, growth defects, and G1 arrest. (A)** β-Galactosidase staining of control and patient obtained fibroblasts each conducted after eight splits from stock (scale bar, 50 μm). **(B)** Quantification of the percentage of cells displaying senescence. **(C)** Growth curve for patient fibroblasts containing POT1(L259S) and control cells. **(D)** Propidium iodide (PI) flow cytometry analysis of control and IPF patient fibroblasts. Data for each panel were obtained from two independent experiments, each performed in duplicate.

We noticed that growth of POT1(L259S) patient fibroblasts declined rapidly compared with healthy fibroblasts containing POT1(WT), as demonstrated in the growth curve of Fig. 9 A. This led us to hypothesize that these cells were entering premature senescence. To address this question, we performed β-galactosidase staining of POT1(L259S) patient and control fibroblasts. The patient and control fibroblasts were grown in parallel to an equal number of passages (eight). At 80% confluence (2.2 × 10^6^ cells on a T-25 plate), the cells were fixed and stained for 48 h before imaging. From analysis of 1,084 control fibroblasts and 1,027 IPF patient cells, we found that POT1(L259S)-containing fibroblasts had a 10-fold increase in senescent cells compared with control fibroblasts (Fig. 9; P < 0.0001). We additionally performed flow cytometry with propidium iodide cell cycle analysis (Crowley et al., 2016). 10,000 cells were assessed to monitor the percentage of patient and control fibroblasts in G1, S, and G2/M phases, respectively. We found that patient fibroblasts were largely arrested in G1 phase, agreeing well with the notion that these cells are largely senescent (Stein and Dulić, 1995). Specifically, we found a 13% increase in G1 cells in L259S fibroblasts compared with control cells, with corresponding reductions in S and G2 phases (Fig. 9 D).

## Discussion

IPF is often associated with a dysfunctional telomerase and in some cases with the shelterin genes TIN2 and TPP1 and the helicase RTEL1 (Hoffman et al., 2016; Antoniou et al., 2013; Armanios, 2012; Armanios et al., 2007; Dai et al., 2015; Dressen et al., 2018; Herrmann, 2008; Le Saux et al., 2013; Tsang et al., 2012; Waisberg et al., 2010; Waisberg et al., 2012; Zheng et al., 2018; Kannengiesser et al., 2015; Du et al., 2018). It is interesting that this novel *POT1* mutation (L259S) results in premature senescent cells, as increased senescent lung cells are a phenotypic sign of IPF (Schafer et al., 2017). Similar phenotypes have been reported when shelterin genes are removed. For example, a conditional deletion of TRF1 in mice caused age-dependent lung remodeling with fibrosis when confined to alveolar type 2 (AT2) cells only and increased numbers of senescent AT2 cells (Naikawadi et al., 2016). In lung tissue of IPF patients, short telomeres were shown to be a characteristic of AT2 cells in fibrotic lesions (Snetselaar et al., 2017). However, short lung telomere length is not a general characteristic of lung tissue of IPF patients. Instead, only a subset of IPF patients (∼50%) have significantly short AT2 telomere length, and the presence of short lung telomeres is associated with short leukocyte telomere length and telomere-related mutations (van Batenburg et al., 2020).

DNA binding assays indicating that POT1(L259S) binds telomeric DNA with reduced affinity (Fig. 2 B) are consistent with the POT1N-DNA structure (Lei et al., 2004). The structure shows that the L259S mutation introduces a polar amino acid into a hydrophobic cluster of residues. The cluster forms part of a hairpin that is critical to the formation of the DNA binding pocket of POT1(OB2) (Fig. 2). The POT1(L259S) mutation likely disrupts the organization of the hairpin, which makes direct contact with the DNA substrate and leads to the observed loss in DNA affinity. POT1 binding to the telomeric overhang is crucial for suppressing ATR-dependent DDR (Kratz and de Lange, 2018). Defects in POT1-DNA binding have been shown to result in uncapped telomeres that are targeted by ATR-dependent DDR and homologous recombination (Tong et al., 2015; Wu et al., 2006).

Because POT1(L259S) is defective in telomeric DNA binding, we were not surprised to find that the POT1 mutant was not localized as prominently to the nucleus as POT1(WT). DNA binding has been shown to be a contributing factor for nuclear accumulation of certain proteins (Sackey et al., 1996). A potentially similar case is seen with DNA binding–defective mutant STAT5B proteins that are impaired in nuclear localization (Herrington et al., 1999). Because fewer POT1 molecules may be bound to DNA at a given moment, perhaps more POT1 is free to leave the nucleus. It is also possible that less POT1(L259S) reaches the nucleus compared with WT. L259S may affect nuclear translocation instead of retention. POT1 has no known NLS signal, but L259S may affect binding of POT1 with another NLS-containing protein, as has been suggested of FOXP2 (Tanabe et al., 2011). It is possible that the cytoplasmic POT1 is transported to lysosomal compartments for degradation, which would explain the witnessed punctate spots seen within the cytoplasm for POT1(L259S) (Fig. 4).

Mutant POT1 has also been implicated in CP and cancer. Recently, two siblings with CP from consanguineous parents were reported to both be homozygous for the *POT1* p.(S322L) mutation (Takai et al., 2016). Unlike IPF, which affects lung tissue, CP patients develop symptoms primarily in the retina as well as the brain, bones, and gastrointestinal tract. The siblings homozygous for *POT1*(S322L) developed symptoms of CP extremely early in life (age 1.5 yr; Takai et al., 2016). This variant of *POT1*(S322L) was found to be defective in negative regulation of telomerase activity, resulting in hyperelongated overhangs and telomere truncations (Takai et al., 2016). We show that the POT1 IPF mutation results in a similar loss in telomere length regulation when introduced to dividing HEK293T cells (Fig. 6) and overall seems to cause similar telomere-related defects. This naturally raises the question of why one POT1 mutation could lead to CP while the other results in IPF. CP is known to occur as the result of mutations in CST (specifically CTC1 and STN1), and only one POT1 mutation has also been implicated in this disease (Takai et al., 2016; Chen et al., 2013). The current hypothesis regarding the mechanism of POT1-associated CP is that this mutant (POT1 S322L) interferes with the interplay between POT1 and CST, which affects telomeric c-strand synthesis. As POT1 S322L is not defective in telomeric binding, the mutation results in similar cellular phenotypes to a CTC1 mutation (K242Lfs*41; Takai et al., 2016). CP is considered an autosomal recessive disease, meaning a condition of homozygosity is required. It is likely that homozygous dysfunctional POT1 associates with the more severe phenotypes of CP, with pediatric presentation and multiorgan involvement, while heterozygous carriage of a pathogenic mutation may first manifest as IPF later in life. Familial pulmonary fibrosis caused by a telomere-related gene mutation is usually an autosomal dominant disease. In families with mutations associated with short telomeres, genetic anticipation (earlier onset of symptoms with successive generations) has been described. IPF is often the first manifestation of disease in mutation-carrying families and occurs in subjects in the sixth or seventh decade of life. Previously we showed that up to seven generations of mutation carriers may pass before disease manifestation occurs (van Batenburg et al., 2020). Moreover, once manifested, disease anticipation occurred in the family (van Batenburg et al., 2020). The pedigree of our IPF patients with the heterozygous *POT1*(L259S) mutation shows considerable and comparable anticipation in both lineages.

*POT1* mutations in the DNA-binding domain have been previously implicated as drivers of cancer (Robles-Espinoza et al., 2014; Calvete et al., 2017; Chen et al., 2017; Kim et al., 2021; Shen et al., 2020a; Shen et al., 2020b; Srivastava et al., 2020; Wu et al., 2020). Many of these are de novo missense mutations that directly affect DNA binding and so may be functionally like POT1(L259S). In our experiments, the fraction of prematurely senescent cells bearing the POT1(L259S) mutation is striking. Senescence is commonly considered to be an endogenous mechanism to prevent cancer (Lynch, 2006). We believe senescence to be the end downstream effect of the following: reduction of binding affinity of POT1 for the single-stranded telomeric overhang, defective nuclear localization, defects in telomeric c-strand (lagging strand) synthesis, and/or the increase in telomere-induced DNA damage resulting from POT1(L259S). It is probable that cancer development in a POT1 mutant may require the presence of mutated oncogenes, such as TP53.

We propose an underlying mechanism of IPF arising from a combination of POT1(L259S)-associated defects including reduced nuclear accumulation, diminished telomeric overhang binding, and inability to downregulate telomerase. Over time, these POT1 defects result in telomere loss (sister loss and telomeric signal free ends), increase in TIFs, and ultimately premature cellular senescence (a hallmark of IPF). Taken together, these results suggest a molecular basis for IPF resulting from a *POT1* variant and pave the way for potential gene therapies seeking to exchange mutant POT1(L259S) for POT1(WT) or interventional drugs seeking to increase POT1 functionality.

### Conclusion

In this study, we show that a family carrying a *POT1* mutation has typical characteristics of telomere syndromes, including (a) mutation-carrying family members have relatively short telomeres, (b) multiple phenotypes of short telomere syndromes are present in the family, and (c) the family shows genetic anticipation (earlier onset of disease in subsequent generations). Furthermore, we show that the mutation is unique (never reported in any database worldwide) and segregates with disease over multiple generations. Experiments with the POT1(L259S) mutation showed reduction in binding affinity to single-stranded telomeric overhangs. POT1(L259S) does not accumulate in the nucleus, and telomeres become uncapped. Uncapped telomeres lead to formation of TIFs as well as short telomeres, which in turn results in premature senescence. Altogether, the data show the deleterious effects of this unique POT1 mutation, providing strong evidence for pathogenicity according to the American College of Medical Genetics and Genomics and the Association for Molecular Pathology published guidelines. Moreover, the study will cause the inclusion of POT1 in clinical genetic pipelines, providing diagnostic and future treatment opportunities for IPF patients.

## Materials and methods

### Protein expression and purification

Human POT1N, consisting of residues 6–299, was cloned into a pET28b vector containing a N-terminal hexahistidine-pMocr fusion tag. The WT plasmid was mutated to contain the L259S variant via site-directed mutagenesis. WT and L259S POT1 were overexpressed in *Escherichia coli* BL21-CodonPlus (DE3)-RIPL competent cells (Agilent Technologies) at 16°C for 16 h using 1  mM IPTG (Gold Biotechnology). The cells were harvested by centrifugation and lysed in a buffer containing 25 mM Tris HCl, pH 7.5, 1.0 M KCl, 1.0 M urea, 5% glycerol, 1 mM PMSF, and 1 mM benzamidine (Ni Buffer A) via sonication. The proteins were purified over a Ni-nitrilotriacetic acid column and buffer exchanged while on the Ni-NTA column with 25 mM Tris HCl, pH 7.5, 0.2 M KCl, and 5% glycerol (Ni Buffer C). POT1N was eluted with 300 mM imidazole onto a HQ (poros) column (Applied Biosystems) equilibrated with Ni Buffer C. The protein was eluted from the HQ column with a salt gradient of 0.2 to 1.0 M KCl. Finally, to remove minor remaining impurities, WT and L259S POT1N was bound to a gravity column with 0.5 ml of Talon metal-affinity resin (Clontech) equilibrated with Ni Buffer C. The clean protein was eluted with 225 mM imidazole. Purified protein was concentrated and run on an SDS-PAGE gel. The concentration was determined using a NanoDrop Spectrophotometer (Thermo Fisher Scientific).

### FP assay

FP DNA binding assays were performed in a reaction buffer containing 20 mM Hepes, pH 7.5, 100 mM KCl, 2 mM MgCl_2_, 1 mM EDTA, 2 mM dithiothreitol, 1 mg/ml BSA, 5% vol/vol glycerol, and 75 nM polyT50 competitor (IDT). The DNA probe containing the 5′-fluorescein tag (6-FAM/5′-AGGGTTAGGGTTAG-3′) was purchased from IDT. POT1N concentration ranged from 0 to 250 nM, and the probe was used at a concentration of 5 nM. Binding reactions were incubated at room temperature for 30 min before 18-μl volumes (in triplicate) were pipetted into a black 384-well OptiPlate (PerkinElmer). Reactions were excited with a 480-nm wavelength and the emissions were measured at 535-nm wavelength using an Envision 2104 Multilabel Plate Reader (Perkin Elmer). The milipolarization values were calculated by the Envision operating software (PerkinElmer). Fitting and binding constants were determined with a one-site binding, nonlinear regression model using PRISM 9 (GraphPad Software).

### Cell culture

Patient-derived skin fibroblasts were obtained from a skin punch biopsy and grown in NUT.MIX.F-12(HAM) w/Glutamax (HAMF-12), supplemented with 10% FBS and penicillin/streptomycin (15140122; Gibco). They were cultured in DMEM (10-013-CV; Corning) supplemented with 1× penicillin/streptomycin (15140122; Gibco) and 10% heat-inactivated FBS (S1620; Biowest) at 37°C and 5% CO_2_. HEK293T were selected with shRNAs targeting POT1 as in our previous publication (Rice et al., 2017). These cells were then introduced with Flag-POT1(WT) or FlagPOT1(L259S) using pLU iBLAST vectors. Cells were selected using growth medium supplemented with 5 μg/ml blasticidin S and 2 μg/ml puromycin.

### Genetic analysis

We isolated patient DNA from peripheral blood samples followed by Exome sequencing (Novaseq 6000 sequencer; Illumina) and data processing at the University Medical Center, Utrecht, Netherlands. After enrichment of the exome with the Agilent SureSelect CREV2kit (Agilent Technologies), whole-exome sequencing was performed on a Novaseq 6000 sequencer (Illumina). Illumina sequencing data was processed with the in-house pipeline, IAP v2.6.1 (Hoffman et al., 2019) and GATK v3.4-46 following the best-practice guidelines (Van der Auwera et al., 2013). We filtered the results for exonic variants with a population frequency <0.5% in 36 genes related to telomere syndromes or pulmonary fibrosis (*ABCA3*, *ACD*, *AP3B1*, *CSF2RA*, *CSF2RB*, *CTC1*, *DKC1*, *FAM111B*, *HPS1*, *HPS4*, *ITGA4*, *LIG4*, *MARS*, *NAF1*, *NKX2-1*, *NOP10*, *PARN*, *POT1*, *RNF168*, *RTEL1*, *SAMD9L*, *SFTPA1*, *SFTPA2*, *SFTPB*, *SFTPC*, *SFTPD*, *STN1*, *TEN1*, *TERC*, *TERF1*, *TERF2*, *TERT*, *TINF2*, *TMEM173*, *USB1*, and *WRAP53*). We measured leukocyte telomere length by quantitative PCR as described previously (Snetselaar et al., 2015). Briefly, telomere length was estimated for each sample from the ratio of telomere repeat copy number to a single gene (human β-globin gene) copy number (T/S ratio). Reference values were derived from a cohort of 164 healthy individuals 20–70 yr of age. The study was approved by the Medical Research Ethics Committees United of St Antonius Hospital (R05-08A), and patients provided written informed consent.

### Metaphase spreads

Cultured fibroblasts were grown to 70% confluency and then treated with fresh culture medium with 0.1 μg/ml colcemid (15212012; Thermo Fisher Scientific) added. Cells were then incubated at 37°C at 5% CO_2_ for 5 h. Following incubation, cells were trypsinized and resuspended dropwise in a 75-mM KCl hypotonic solution. Cell suspensions were then incubated at 37°C for 20 min with occasional end-to-end gentle mixing. After this incubation, cells were immediately fixed in 3:1 methanol acetic acid dropwise three times with centrifugation at 1,000 rpm conducted in between each fixation step. Fixed cells were then dropped onto prechilled, moist glass microscope slides. Cells were then allowed to dry at 40°C overnight. The next day, the slides were rehydrated with PBS for 2 min twice. After hydration, the cells were then fixed again to the slides using a 4% freshly prepared formaldehyde solution in PBS for 5 min. Slides were then rinsed twice with PBS and incubated in a pepsin solution (0.005% pepsin in 10 mM HCl) for 5 min to aid in permeabilization. Slides were then rinsed twice with PBS, dehydrated in 70, 85, and 100% ethanol for 2 min each, allowed to air dry, and preheated to 85°C while a hybridization solution was freshly prepared (60% formamide, 0.5% blocking reagent [11096276001; Roche], and 20 mM Tris, pH 7.4). PNA-FISH telomeric probes were added to the hybridization solution (PNA-Bio), and this was also preheated to 85°C. 50 μl of the probe solution was then dropped onto each slide, and a coverslip was immediately placed on top. The slide was then heated at 85°C for 10 min, after which hybridization was allowed to occur for 2 h at room temperature in the dark. After hybridization, the slides were washed three times in 2× SCC with 0.1% Tween at 60°C. Slides were then counterstained with DAPI for nuclear visualization and finally imaged at 100× using a Nikon 80i microscopic setup.

### Western blotting and coimmunoprecipitation

All lysates were generated using CHAPS lysis buffer (150 mM KCl, 50 mM Hepes, pH 7.5, 1 mM MgCl_2_, 1 mM EDTA, 10% glycerol, 0.5% CHAPS [3-[(3-cholamidopropyl)dimethylammonio]-1-propanesulfonate], 5 mM β-mercaptoethanol, and 0.01 μl/ml protease inhibitor cocktail [P8340; Sigma-Aldrich]). For probing, anti-Flag (14793S; Cell Signaling Technology) was used at 1:1,000 dilution. For endogenous controls, anti-GAPH was used at 1:1,000 (5174T; Cell Signaling Technology). Coimmunoprecipitations were performed using anti-Flag agarose beads (M8823; Sigma-Aldrich). In brief, HEK293T lysates containing Flag-POT1 and HA-TPP1 were incubated in BC150 buffer (25 mM Hepes, 2 mM EDTA, 150 mM KCl, and 10% glycerol, pH 7.6) with 20 μl of Flag beads for 6 h at 4°C. Beads were then washed with lysis buffer and BC150 three times. Elution was performed with 0.3 mg/ml Flag peptide. Western blots were run on elutions and probed for Flag and hemagglutinin (HA). Anti-HA was used at 1:1,000 (3724T; Cell Signaling Technology).

### Interphase telomere-FISH

For interphase telomere-FISH experiments, fibroblasts were grown directly onto sterilized glass slides and fixed in freshly prepared 4% formaldehyde in PBS. Slides were then incubated in 70% ethanol until ready for hybridization steps. Hybridization was conducted as described for metaphase spreads, except leading and lagging strand probes were added in a sequential manner. In brief, the leading strand probe was added to slides in hybridization buffer at 85°C for 5 min at a concentration of 500 nM. The slides were then washed three times in hybridization buffer to remove unbound probe at 60°C. Then the lagging strand probe was added in the same manner. For probing the leading strand, a Cy3 dye–labeled PNA probe was used, and for the lagging strand, an Alexa Fluor 647 dye–labeled PNA probe was used. Both probes were obtained from PNA-Bio. Imaging was conducted at 100× or 40× using a Nikon 80i microscope setup with appropriate filter sets. Leading and lagging strand quantification of telomeric spots was conducted using Fiji (ImageJ) analyze particles function (Mesquita et al., 2020).

### Immunofluorescence and YFP POT1 imaging

Fibroblasts were grown directly onto sterilized glass slides and fixed in freshly prepared 4% formaldehyde in PBS for 10 min. Slides were then incubated in 70% ethanol until ready for immunofluorescence. Slides were washed twice with PBS and incubated for 30 min in blocking solution (sc-516214; Santa Cruz Biotechnology). Slides were incubated with anti-POT1 (A1491; Abclonal) at a dilution of 1:100 in blocking solution for 1 h. For assessing DNA damage foci, an anti-53BP1 antibody (4937S; Cell Signaling Technology) was used. Slides were washed three times for 5 min in PBS. A Santa-Cruz 647–labeled secondary antibody (sc-516251) was then used at a dilution of 1:200 and incubated with slides for 1 h. Slides were again washed three times for 5 min in PBS. Slides were dehydrated and treated for FISH as described above for metaphase spreads and chromosome orientation FISH. Finally, cells were counterstained with DAPI and imaged at 100× or 40× on a Nikon 80i setup. Nuclear/cytoplasmic ratios were calculated using the ImageJ plugin Intensity_Ratio_Nuclei_Cytoplasm.ijm as in previous publications (Kelich et al., 2021). For HEK293T cells expressing YFP-POT1, the same microscope setup and analysis techniques were used, but YFP filter sets were used instead of Alexa Fluor 647 filter sets. Cells were grown on microscope slides, fixed in 4% formaldehyde, and counterstained with DAPI before imaging.

### Confocal imaging

POT1 knockdown cells as in our previous publication (Rice et al., 2017) were transfected with N-terminal YFP WT or L259S POT1 as well as mCherry TRF2. Cells were then grown on microscope slides, fixed in 4% fresh paraformaldehyde, and counterstained with DAPI. Slides were then used for taking confocal images measuring mCherry, YFP, and DAPI on a Leica TCS SP8 white light laser scanning confocal microscope. 1 μm Z slices were processed using ImageJ software.

### Telomere overhang analysis

Telomere overhang analysis was performed as described in our previous manuscript (Shastrula et al., 2018). In summary, pure genomic DNA was isolated from cells using a GeneJET purification kit (Thermo Fisher Scientific). 15 μg was then digested with ExoI (New England Biolabs) overnight. The next day, AluI and MboI (New England Biolabs) were added to the reaction, and 15 μg of control (non–ExoI treated) DNA was also digested. DNA was then purified through ethanol precipitation and run on a 0.7% large agarose gel. The gel was dried using a vacuum gel drier (Bio-Rad) for 30 min, and the dried gel was hybridized with a ^32^P-labeled (5′-CCCTAA-3′)4 probe in Church buffer overnight and at 42°C. The gel was washed twice with 0.25× SSC (a ready-to-use saline sodium citrate buffer, pH 7.0–7.5, that contains 0.3% NP40 and Proclin 950 as a preservative) with 0.1% SDS and then 0.25× SSC for 30 min each. The gel was exposed to a phosphor-imager (GE Healthcare) and visualized with a Typhoon RGB Imager (GE Healthcare). To reprobe the gel for total telomeric signal, the gel was denatured in alkaline solution (0.5 M NaOH and 0.15 M NaCl) twice for 30 min each and neutralized in 0.5 M Tris-HCl, pH 7.5, and 3 M NaCl twice for 20 min each. The gel was preequilibrated in 5× SSC for 15 min and hybridized as above with a ^32^P-labeled (5′-CCCTAA-3′)4 probe.

### Cell cycle analysis

Cell cycle analysis was conducted using propidium iodide staining and flow cytometry. 1 million cells for each condition were collected, pelleted, and washed twice with PBS. Cells were then fixed in ice-cold 70% ethanol for ≥30 min. Cells were treated with RNase A (100 μg/ml) for 5 min and stained with a 1:500 dilution of propidium iodide at 10 mg/ml in PBS. The cells were flowed through a LSRII 14-color flow cytometer to screen for propidium iodide signal. 10,000 events were used for each graph.

### Direct telomerase activity assays

Direct telomerase activity assays were performed as described by Cristofari and Lingner (2006) and our previous publication (Rice et al., 2017). In summary, HEK293T cells knocked down from endogenous POT1 were transfected with pcDNA6-hTERT and pBS-U1-hTER (plasmids were a gift from J. Lingner, École Polytechnique Fédérale de Lausanne, Lausanne, Switzerland). 1 μg of TERT plasmid and 3 μg of TER plasmids were used with Lipofectamine 2000 transfections. For POT1 and TPP1 experiments, 1 μg of pLU Flag-POT1 (WT or L259S) and HA-TPP1 plasmids were introduced as well. Lysates from T25 flasks were generated in 300 μl of CHAPS lysis buffer consisting of 150 mM KCl, 50 mM Hepes, pH 7.5, 1 mM MgCl_2_, 1 mM EDTA, 10% glycerol, 0.5% CHAPS, 5 mM β-mercaptoethanol, and 0.01 μl/ml protease inhibitor cocktail (P8340; Sigma-Aldrich). 5 μg lysate was used to carry out the primer extension reactions using 20 nM A5 primer (5′-TTA​GGG​TTA​GCG​TTA​GGG-3′). Primer extension was allowed to occur for 1 h at 30°C in buffer containing 50 mM Tris HCl, pH 8.0, 50 mM KCl, 1 mM spermidine, 1 mM MgCl_2_, 5 mm β-mercaptoethanol, 500 μM dATP, 500 μM dTTP, μM dGTP, and 20 μCi of [α-^32^P]dGTP (3,000 Ci/mmol). Reactions were stopped, and DNA was precipitated with 100 μl of 3.6 M ammonia acetate, 20 μg glycogen, and 0.5 nM 5′ ^32^P-labelled 18mer loading control. Samples were left at −80°C overnight. Samples were then spun down for 30 min at 14,000 rpm, and ethanol was removed. Pellets were washed with 70% ethanol to remove excess nucleotides. The pellets were air dried and resuspended in 98% formamide, 1 mM EDTA, and 0.05% xylene cyanol; heated to 95 °C for 5 min; and loaded onto a 10% acrylamide, 8 M urea, 0.5× TBE sequencing gel. The gel was run for 2.5 h at 1,800  V, fixed with 30% methanol/10% acetic acid solution, and dried for 1 h with a gel dryer at 80°C (Bio-Rad). The dried gel was exposed to a phosphor screen overnight and imaged the next day with a Typhoon RGB Imager (GE Healthcare).

### Senescence staining

IPF patient fibroblasts containing the L259S mutation were cultured in parallel with normal diploid fibroblasts (CCD-1058Sk; American Type Culture Collection CRL-2071) to an equal number of passages. Cells were stained for 48 h using a senescence kit (Cell Signaling Technology).

## Supplementary Material

SourceData F2 contains original blots for Fig. 2.

SourceData F3 contains original blots for Fig. 3.

SourceData F6 contains original blots for Fig. 6.
